# MEMe: A Mutually Enhanced Modeling Method for Efficient and Effective Human Pose Estimation

**DOI:** 10.3390/s22020632

**Published:** 2022-01-14

**Authors:** Jie Li, Zhixing Wang, Bo Qi, Jianlin Zhang, Hu Yang

**Affiliations:** 1 Key Laboratory of Optical Engineering, Chinese Academy of Sciences, Chengdu 610209, China; lijie163@mails.ucas.edu.cn (J.L.); 202111012048@std.uestc.edu.cn (Z.W.); jlin@ioe.ac.cn (J.Z.); yangh@ioe.ac.cn (H.Y.); 2 Institute of Optics and Electronics, Chinese Academy of Sciences, Chengdu 610209, China; 3 University of Chinese Academy of Sciences, Beijing 100039, China; 4 School of Information and Communication Engineering, University of Electronic Science and Technology of China, Chengdu 610209, China

**Keywords:** human pose estimation, deep learning, mutually enhanced, efficient and effective, modeling method, extended convolutions, feature fusion, attention mechanisms, CNN

## Abstract

In this paper, a mutually enhanced modeling method (MEMe) is presented for human pose estimation, which focuses on enhancing lightweight model performance, but with low complexity. To obtain higher accuracy, a traditional model scale is largely expanded with heavy deployment difficulties. However, for a more lightweight model, there is a large performance gap compared to the former; thus, an urgent need for a way to fill it. Therefore, we propose a MEMe to reconstruct a lightweight baseline model, EffBase transferred intuitively from EfficientDet, into the efficient and effective pose (EEffPose) net, which contains three mutually enhanced modules: the Enhanced EffNet (EEffNet) backbone, the total fusion neck (TFNeck), and the final attention head (FAHead). Extensive experiments on COCO and MPII benchmarks show that our MEMe-based models reach state-of-the-art performances, with limited parameters. Specifically, in the same conditions, our EEffPose-P0 with 256 × 192 can use only 8.98 M parameters to achieve 75.4 AP on the COCO val set, which outperforms HRNet-W48, but with only 14% of its parameters.

## 1. Introduction

**Figure 1:**
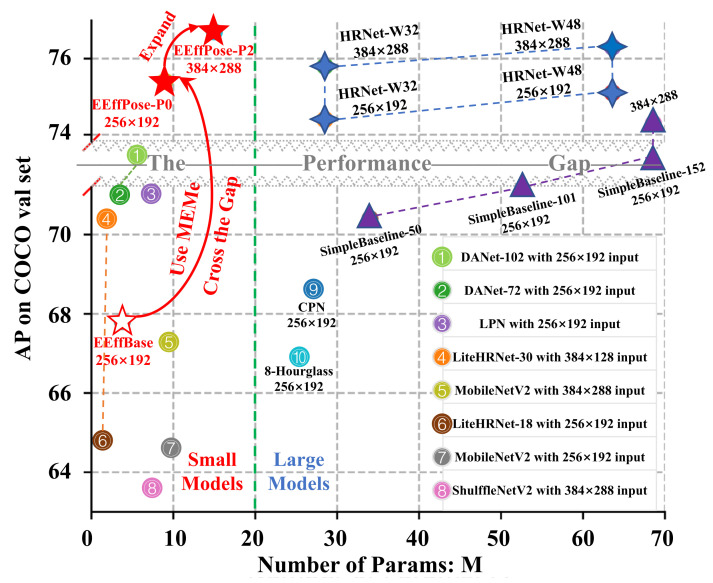
Demonstration of the performance gap (in gray) and comparison of our models (in red) with others. In the figure, the model with less parameters numbers and higher AP is more efficient and effective, respectively. Efficient baseline (EffBase) means our baseline model transformed from EfficientDet. While efficient and effective pose (EEffPose) is reconstructed from EffBase by using our mutually enhanced modeling method (MEMe). It is noteworthy that our MEMe-based models (EEffPose-P0 and its expanded version EEffPose-P2), cross the performance gap, archive the state-of-art among small models, and even surpass HRNet—the typical representative of large models.

Since 2016, deep learning-based methods [1,2] have become a prime focus of research in 2D human pose estimation, greatly promoting the development of action recognition [3] and other human-centered applications [4,5]. Those deep learning models can be categorized as large models with high performances and small models with low accuracies, leading to a performance gap in Figure 1. To fill this gap between the complicated and lightweight models, this paper explores a general modeling method used to make the lightweight models “cross the gap”, i.e., with better performances than the big ones.

Traditionally, to overcome the challenges in the scale variances and keypoint occlusions, various classic large models are proposed, such as stacked hourglass [6], CPN [7], SimpleBaseline [8], and HRNet [9]. Stacked hourglass consists of multiple stacked hourglass-shaped modules with intermediate supervision, which is the first multi-scale representation network architecture in human pose estimation, but is complex and inefficient. To solve this problem, CPN cascades only two pyramid nets with ResNet as the backbone, where one is a global net and the other is a refine net, to make better auxiliary supervision. SimpleBaseline directly proposes a single-stage hourglass, where the features are downsampled by the ResNet to encode information, and then upsampled by deconvolution to decode the final output. As for HRNet, it is designed to maintain high-resolution representation by multi-scale parallel branches, which is more efficient and effective than ever, but still has largely redundant parameters and high complexity, and is not suitable for real deployment. In short, large models still need to be more efficient.

Recently, to gain a lightweight model with high accuracy, the main logic has been to use the single-stage architecture, and follow efficient models, such as MobileNet [10,11,12] and ShuffleNet [13,14]. A MobileNet-based method [15] generally upsamples the original convolution layer resolution and adds skip connections, but cannot remain a high-resolution representation, which could lead to poor performance. ShuffleNet-based methods, such as LiteHRNet [16], use the channel split–shuffle operation to reduce model complexity, which loses representation flexibility and model performance. In addition, the latest DANet [17] uses well-designed blocks with orthogonal attention and some special post-processing techniques to achieve the state-of-art among small models, but it still cannot cross the performance gap between large models and small models in Figure 1. Thus, small models need to be more effective.

**Figure 2:**
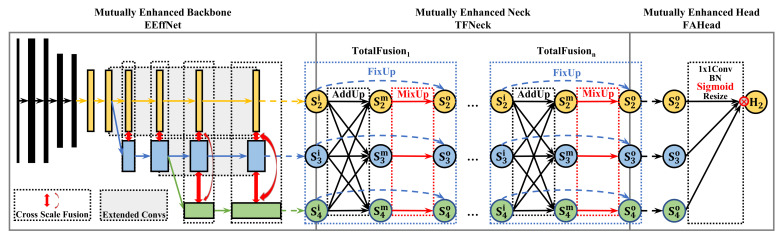
An overview of our MEMe-based pose net, efficient and effective pose (EEffPose). Our EEffPose contains three modules: enhanced EfficientNet (EEffNet), total fusion neck (TFNeck) and final attention head (FAHead). In figure, $S_{j}$ means the j-th scale feature in the feature pyramid. In particular, $H_{2}$ represents the heatmap output with the size of a quarter of the input.

In this paper, we propose a mutually enhanced modeling method (MEMe) to mutually enhance a lightweight model performance, but with low complexity. We transferred EfficientDet (EffDet) [18] into a human pose estimation to form an efficient human pose baseline named EffBase, by combining the following three modules: EfficientNet (EffNet), the original backbone of EffDet; BiFPNNeck, the original neck of EffDet; and final sum head (FSHead) commonly used for human pose estimation. Then, we used MEMe to reconstruct it into an efficient and effective pose net (EEffPose), as shown in Figure 2. Similarly, it contains three mutually enhanced modules: the MEMe-based backbone, efficient and effective net (EEffNet); the MEMe-based neck, total fusion neck (TFNeck); and the MEMe-based head, final attention head (FAHead).

For the backbone, EffNet is mutually enhanced into EEffNet, where the MEMe means the extended convolution and cross-scale fusion. In this way, it can extract more diverse multi-scale features, mutually merging cross-scale information in a more flexible mechanism than before, avoiding the limited and isolated multi-scale feature representations.

For the neck, BiFPNNeck is mutually enhanced into TFNeck by enhancing the repeating BiFPN blocks into total fusion blocks, where a single total fusion block includes three operations: AddUp, MixUp, and FixUp. It is a more sufficient and flexible feature fusion structure that can extract more powerful features and avoid weight-unbalanced problems compared with unidirectional CPN and bidirectional BiFPN.

For the head, FSHead is mutually enhanced into FAHead by using point-wise multiplication instead of sum fusion after the nearest upsampling. Specifically, the FAHead can be regarded as a cross attention mechanism where multi-scale outputs use point-wise multiplication to query and calibrate every corresponding pixels after upsampling instead of gathering together, avoiding the error accumulation due to nearest upsampling and the invalid fusion of information in different scales.

Our contributions are manifold:

- We propose a MEME-based backbone (EEffNet) mutually enhanced by extended convolutions and cross-scale fusions, which bring a comprehensive multi-scale structure to extract more powerful features.
- We propose a MEME-based neck (TFNeck) mutually enhanced by repeating several total fusion blocks containing AddUp, MixUp, and FixUp operations for all scales, which ensure the sufficient, flexible, and weight-balanced information flow.
- We propose a MEME-based head (FAHead) mutually enhanced by Sigmoid and point-wise multiplication to query and calibrate multi-scale outputs, which take full advantage of the multi-branch structure and improve the final output.
- By using MEMe, our EffBase crosses the performance gap and transforms into an efficient and effective pose net (EEffPose), which is superior, with less complexity than state-of-the-art (COCO [19] and MPII [20]) benchmarks. As shown in Figure 1, our EEffPose-P0 with 256 × 192 can use only 8.98 M parameters to achieve 75.4 AP on the COCO-val set, which outperforms HRNet-W48, but with only 14% of the cost.

## 2. Related Works

### 2.1. Efficient Backbone

Backbones like VGG, Hourglass, ResNet and HRNet are always used in human pose estimation. As for VGG backbone, OpenPose [21,22] uses VGG-18 to extract features and PAF to combine body keypoints. As for Hourglass backbone, Stacked Hourglass [6] propose a hourglass-shape encoder-decoder, Hourglass, to get heatmap output, which is used widely as common backbone such as in the AlphaPose [23]. As for ResNet backbone, [24] and CPN [7] use ResNet as backbone and decode the heatmap as the Hourglass while SimpleBaseline [8] uses several deconvolution layers firstly after the ResNet backbone to recover the output. As for HRNet backbone, a multi-scale but high-resolution representation is introduced by [9] firstly and then designed efficiently by [25].

However, to get a lightweight and efficient backbone, some efficient modeling methods were introduced in MobileNet [10,11,12], ShuffleNet [13,14], and EfficientNet [26]. Based on MobileNet’s depth-wise convolutions, DANet [17] proposes the OAB and SFU block to realize an efficient pose model. While using the channel shuffle operation in ShuffleNet, LiteHRNet [16] designs a lite block for a multi-scale model, reducing the complexity, but with a lot of loss in accuracy. EfficientNet—as an efficient backbone—is directly used in EfficientPose [27] for backbone, but fails to get acceptable accuracy.

### 2.2. Feature Fusion Neck

The feature pyramid network (FPN) [28] is the basic top–down information fusion neck, including many variants: PANet [29] adds another bottom–up information-flow layer; NAS-FPN [30] automatically generates the connection relationship among nodes; BiFPN [18] reduces some redundant connection and retains the top–down then bottom–up fusion path to fuse multi-scale features bidirectionally.

In 2D human pose estimation tasks, FPN-like fusion necks are the most common ones where CPN [7], MSPN [31], and Hourglass [6] fuse the low-level features and high-level features together, but only by an unidirectional information flow. There is no FPN-like fusion neck in HRNet [9] leading to the unbalanced feature extraction and the insufficient information fusion.

### 2.3. Final Output Head for Pose Estimation

In general, there are two categories of the final output head for pose estimation: regression head and heatmap head. The former directly regresses the mapping between the input image and coordination of joints [32]. The latter aims to recognize the joints at every pixel of the heatmap [33], which is widely used because of better results than the former. For example, the final sum head in HRNet is a heatmap head used to gather the multi-scale heatmaps as the final output.

Attention mechanisms in transformers [34] are now becoming more popular in a variety of fields, such as classification, objection detection, and target tracking [35]. Among them, DETR [36] reconstructs original feature information by using Q,K,V in detection, first, which is closely related to the field of pose estimation. Affected by this, TFPose [37] uses a transformer while PRCT [38] uses cascaded transformers as the attention regression head to detect the keypoints, but still cannot get a better result, with more parameters and complexities than HRNet. As for the attention heatmap head, polarized self-attention [39] uses the polarized mechanism to get the polarized channel and spatial attention to refine the output, while leading to the increase of complexity.

## 3. Methodology

We first transfered the EfficientDet [18] into an efficient baseline (EffBase) for human pose estimation, which is made up of original EffNet backbone, original BiFPN neck, and final sum head (FSHead). Then we used the mutually enhanced modeling method (MEMe) to enhance it into a brand-new efficient and effective pose (EEffPose) net with the enhanced EffNet (EEffNet), total fusion neck (TFNeck), and final attention head (FAHead), as shown in Figure 2. We presume that our method can succeed in improving the performance of the lightweight model, with low complexity. The formula is shown below:

(1) $$\begin{array}{cc} EEffPose(\cdot )=MEMe(EffBase(\cdot )) & \\ EffBase(\cdot )=FSHead(BiFPN(EffNet(\cdot ))) & \\ EEffPose(\cdot )=FAHead(TFNeck(EEffNet(\cdot ))) \end{array}$$

where EffNet, BiFPN and FSHead are the original backbone, neck, and head of EffBase, respectively. Meanwhile, EEffNet, TFNeck. and FAHead are the MEMe-based backbone, neck, and head of EEffPose, individually.

### 3.1. Enhanced EfficientNet

To make the optimal trade-off between the performance and model complexity, EffNet [26] is introduced as the original backbone, which is a high-efficiency model using the compound expansion method and neural architecture search technology to expand the model’s width, depth, and resolution. It is constructed efficiently by MobileBlock with outstanding performance and low model complexity. However, it cannot be directly used as the backbone here as multi-scale and high-resolution features are critical for human pose estimation.

For the backbone, we apply the MEMe to enhance the EfficientNet by adding extended convolutions and cross-scale fusions to get the enhanced EfficientNet (EEffNet). The extended convolutions enhance the backbone’s multi-scale representation while the cross-scale fusion mutually integrates the multi-scale features further, which transfer the VGG-style backbone into the mutually enhanced HRNet-style backbone. Compared with the former, the latter has more powerful feature extraction capability and high-resolution retaining ability, with multi-scale branches and cross-scale enhancement.

**Figure 3:**
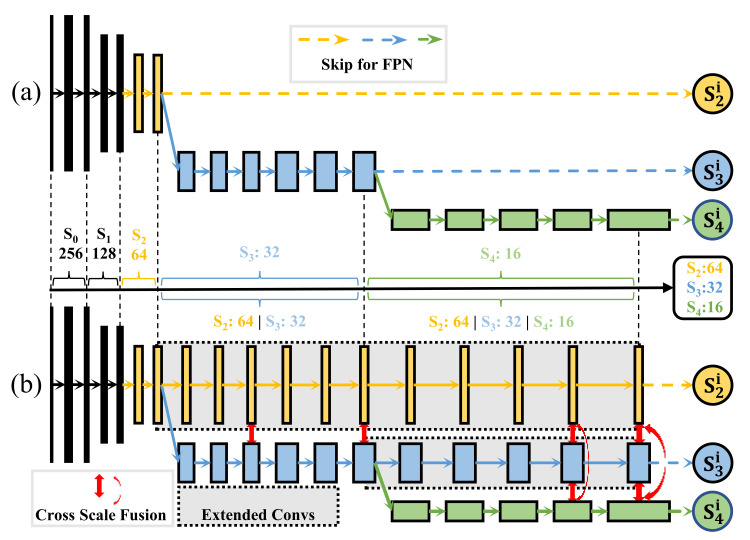
EffNet vs. MEMe-based EEffNet. (**a**) The EffNet. (**b**) The mutually enhanced EEffNet. Compared with (**a**), (**b**) has a more powerful multi-scale representation by using our Extended Convs (gray box), and a more flexible information flow by using our Cross-scale fusion (red arrow). As a MEMe-base backbone, our EEffNet shows how to use MEMe to mutually enhance a backbone.

As shown in Figure 3, compared with EffNet’s one-scale structure, EEffNet is a multi-scale architecture where the extended Convs of EEffNet are cloned from the last convolution of the corresponding scale and the cross-scale fusion represents the total fusion layer, which will be introduced in the next subsection. For example, in the final stage, $S_{2}$/$S_{3}$ (scale with sizes of 64/32), the features go through some extended convolutions, while $S_{4}$ (the scale with size of 16) are dealt by the same convolutions as the original EffNet, then they are integrated by the total fusion layer. By applying MEMe like this, our EEffNet is rebuilt from EffNet, which has a more powerful feature pyramid output.

### 3.2. Total Fusion Neck

**Figure 4:**
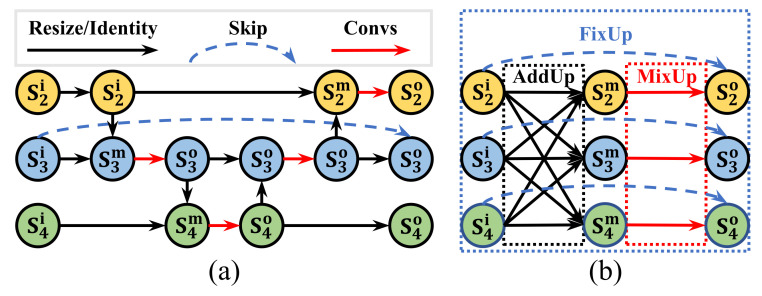
BiFPN vs. MEMe-based TotalFusion. (**a**) the BiFPN, Bidirectional FPN. (**b**) The mutually enhanced TotalFusion. For TotalFusion, the feature pyramid from the backbone is mutually enhanced by AddUp (black), MixUp (red), and FixUp (blue), where ’total’ is the key point compared with BiFPN. As a MEMe-base neck, our TotalFusion shows how to use MEMe to mutually enhance a neck.

Bidirectional FPN (BiFPN) from EfficientDet is a classical multi-scale feature fusion structure in object detection and it inspired us to design an N–N neck structure (N–N means that the input features of the N scales fuse into the output features of the same N scales), which have never been used in human pose estimation before. As shown in Figure 4a, BiFPN can realize the top–down and bottom–up feature fusion, where the consecutive and bidirectional path causes partial, restricted, and weight-unbalanced feature fusion.

Based on this, we propose a total fusion block where the feature pyramid from the backbone is mutually enhanced by a more sufficient, flexible, and a weight-balanced fusion strategy, including AddUp, MixUp, and FixUp. The main idea of the total fusion block is the connection relationship reflected by AddUp, MixUp, and FixUp rather than some magical operations. For example, AddUp emphasizes the need for dense connections between multi-scale features instead of unbalanced connections, such as BiFPN; MixUp is responsible for feature fusion and extraction, to get deeper representations; FixUp connects original-scale features to fix it. Finally, we cascaded the same numbers of repeated total fusion blocks as the number of BiFPN in EffBase to form the total fusion neck (TFNeck).

Formally, the equation of Total Fusion is:

(2) $$\begin{array}{cc} s_{i}^{out} & =TF\left(S_{J}^{in},s_{i}^{in}\right),J=\left\{AllScales\right\}\\ & =FixUp(MixUp\left(AddUp\left(S_{J}^{in}\right)\right),s_{i}^{in}) \end{array}$$

where $s_{i}^{in}$ is *i*-th scale’s input, $S_{J}^{in}$ is the total set of $s_{i}^{in}$, $s_{i}^{out}$ is the *i*-th scale’s output, and *J* means all scale features. Moreover, they can be shown formally below:

(3) $$\begin{array}{cc} s_{i}^{add} & =AddUp\left(S_{J}^{in}\right),J=\left\{AllScales\right\}\\ & =Mish(\sum_{j\in J}w_{j}\cdot {Resize}_{j\to i}\left(s_{j}^{in}\right)) \end{array}$$

(4) $$s_{i}^{mix}=MixUp\left(s_{i}^{add}\right)={MobileBlock}_{i}\left(s_{i}^{add}\right)$$

(5) $$s_{i}^{out}=FixUp\left(s_{i}^{mix},s_{i}^{in}\right)=s_{i}^{mix}+s_{i}^{in}$$

where $s_{j}^{in}$ is *j*-th scale’s input of TF, $s_{i}^{add}$ is *i*-th scale’s output of AddUp and also the input of MixUp. Mish is the activation function. Similarly, $s_{i}^{mix}$ is *i*-th scale’s output of MixUp and the input of FixUp, $S_{J}^{in}$ is the all of $s_{j}^{in}$, and ${Resize}_{j\to i}$ means resize *j*-th to *i*-th scale.

Combined with Figure 4, we explain our equation Equation (2), specifically in terms of the scale’s total fusion. First, for the AddUp (black arrow in Figure 4) operation of Equation (3), there are three scale inputs $s_{j}^{in},j\in \left\{2,3,4\right\}$, where all scale features are added up totally into each output of scale $i_{th}$, which is written as $s_{i}^{add}$. Then, for the MixUp (red arrow in Figure 4) operation of Equation (4), the input $s_{i}^{add}$ independently passes a MobileBlock to get the output $s_{i}^{mix}$. Finally, the FixUp (blue dotted arrow in Figure 4) operation of Equation (5) is an identity shortcut of *i*-th scale, which can ensure input and output feature resolution aligned, as well as keep a smoother gradient flow. To be specific, we extended the skip-connection operation in each scale $s_{i}^{in}$ to add the middle fusion output $s_{i}^{mix}$ into final $s_{i}^{out}$. By applying MEMe on BiFPN like this, our TFNeck can achieve a more sufficient, flexible, and weight-balanced feature fusion.

### 3.3. Final Attention Head

**Figure 5:**
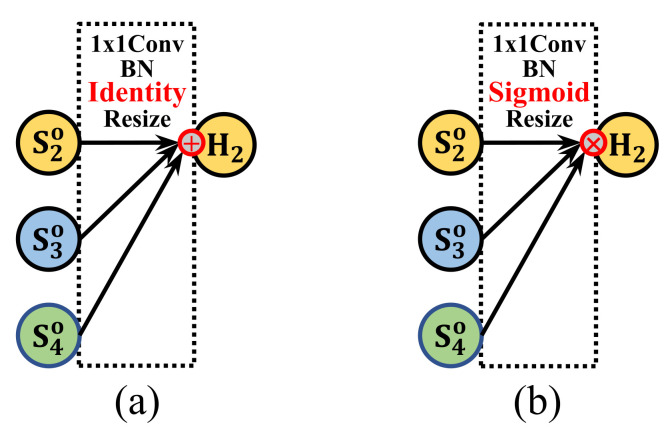
FSHead vs. MEMe-based FAHead. (**a**) Final sum head. (**b**) The mutually enhanced final attention head. The FAHead’s output is mutually enhanced by the Sigmoid and point-wise multiplication to query and calibrate each other, which is a simple but solid way of cross-attention. As a MEMe-base head, our FAHead shows how to use MEMe to mutually enhance a head.

As shown intuitively in Figure 5, the way the FSHead gets the final output by summing or averaging the multi-scale outputs, may introduce the upsampling artificial noises. It may be an inappropriate cross-scale output ensemble by the linear combinations as they are in different semantic level outputs and could not achieve a better consistent output if the network effectively extracts diverse multi-scale information.

Therefore, combined with the attention mechanism rather than the liner combination ensemble, we used the Sigmoid function to activate both the high-level scale feature with more semantic information and the low-level scale feature usually with more detailed information to get multi-scale attention. Then, we point-wise multiply them together to make those attentions query and calibrate each other’s output, which will make better use of their inherent structural advantages in multi-scale receptive fields and circumvent the accumulation of cross-scale errors. After the final attention head, only the heatmap of the low-level scale validated by higher-level attention can be responded to, which means the output is still a low-level scale and high-resolution heatmap without upsampling noise, but with the help of higher-scale attention.

For clear comparison, their formulas can be written as:

(6) $$H_{2}=FSHead\left(S_{J}^{out}\right)=\sum_{j}{ConvBN}_{j}\left(s_{j}^{out}\right)$$

(7) $$H_{2}=FAHead\left(S_{J}^{out}\right)=\prod_{j}\sigma \left({ConvBN}_{j}\left(s_{j}^{out}\right)\right)$$

where input of FAHead $S_{J}$ is the universe of outputs by the previous neck, $H_{2}$ means the output of FAHead, and σ represents the activation function of Sigmoid.

Equation (6) means the traditional sum fusion that directly fuses the features from the different feature spaces and is harmful for location information representation. By applying a MEMe like this, our Equation (7) utilizes the Sigmoid activation function and Point-Wise Multiplication to realize the cross attention of the $S_{J}$ leading to a calibrated output.

### 3.4. Instantiation

In this paper, to make a fair comparison, we used MEMe to reconstruct the EffBase into EEffPose-P0 for 256 × 192 and EEffPose-P2 for 384 × 288 on COCO. For the detailed structure information, EEffPose-P0/P2’s backbone EEffNet-B0/B2 were transferred from EffNet-B0/B2. As for the TFNeck of EEffPose-P0/P2, the feature pyramid of EEffNet-B0/B2 go through the same number of repeated total fusion blocks as EfficientDet-D0/D2. As for the head of EEffPose-P0/P2, they are sampled to the corresponding keypoint heatmap, whose size is the same as $S_{2}$ by convention.

Finally, our EEffPose-P0/P2 has the parameters of 8.98 M/16.7 M, and a GFLOPs of 4.94 G/23.0 G. Moreover, compared with HRNet-W32/W48, whose parameters are 28.5 M/63.6 M, and GFLOPs are 7.7 G/35.4 G, our MEMe-based models are of low-cost in the expanded deployments.

## 4. Experiments and Analysis

**Figure 6:**
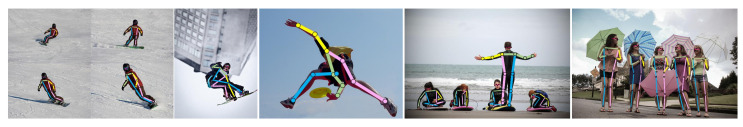
Presentation for COCO: including viewpoint change, occlusion, and multiple persons.

**Figure 7:**
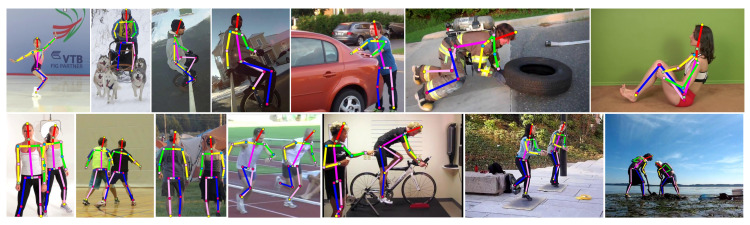
Presentation for MPII: including viewpoint change, occlusion, and multiple persons.

In this paper, we used MS COCO and MPII as the evaluation benchmarks to show the performance of our MEMe-based EEffPose and to verify the necessity of MEMe. To investigate the effectiveness of specific modules of the model and demonstrate the versatility of MEMe, we designed the ablation study on COCO val and conducted a series of visualizations for further explanation. Then, by comparing with the state-of-the-art, including small and large models, our MEMe-based EEffPose showed a superior performance and the best trade-off between performance and complexity. Finally, we demonstrated some qualitative presentations for samples in COCO and MPII, as shown in Figure 6 and Figure 7.

Implementation Details. Our experiments were trained on 4 NVIDIA A100 GPUs with PyTorch 1.9, CUDA 11.1, and cuDNN 8.0.5. For the system, it was a Ubuntu 16.04 running on an Intel E5-2678v3@2.50 GHz CPU and 64 G RAM memory.

Datasets. The COCO dataset [19] is a rich dataset containing more than 90 targets, 0.3 billion images, and 2.5 billion labels. We trained our model on a MS COCO 2017 training set, which included 57,000 images and 150,000 person instances with 17 keypoints. The validation set including 5000 images was used to validate training performance. The results on the test-dev set (20,000 images) were also list, to make a more fair and convincing comparison between our model and other SOTA methods. Moreover, we report results on the MPII dataset [20]. MPII is a popular benchmark for single-person 2D pose estimation, which has 25,000 images. In total, there are 29,000 annotated poses for training, and another 7000 poses for testing.

Training. In the training process, each GPU occupies 16 samples. The Adam optimizer is used with ReduceLROnPlateau, where the initial learning rate is 0.001, the descending factor is 0.3, and descending patience is 5. Augmentation operations are used for each input image, including scale, rotation, flipping, half body data augmentation (only for COCO), and cropping, which is the same as HRNet [9] and SimpleBaseline [8]. The scaling factor is sampled between ±0.35/±0.30 and the the rotating factor is between ±45°/±25° for COCO and MPII, respectively. Finally, the cropping operation gets a size of 256/384 for EEffPose-P0 and EEffPose-P2, while keeping the aspect ratio to 3:4/4:4 for COCO and MPII.

Testing. Testing is a top–down process, where the human target is extracted first and then keypoints are detected. Moreover, this testing process is the same as the HRNet and SimpleBaseline. The model outputs a heatmap where all keypoint detected results and flipped image results are averaged. Finally, the final position is achieved by a quarter of the pixel’s shift from the main wave peak.

Evaluation. Mean average precision (mAP) from object keypoint similarity (OKS) is used as the evaluation metric on COCO, where OKS uses the Euclidean distance between the predicted keypoints and ground-truths to evaluate the similarity of keypoint pairs.The head-normalized probability of correct keypoint (PCKh) is the evaluation metric on MPII, which can detect whether the keypoints locate in the ground-truth adjacent range. In our paper, we demonstrate the outcomes of AP, AP .5 (IOU > 0.5), AP .75 (IOU > 0.75), AP (M) (for the middle targets), AP (L) (for the large targets), and AR on COCO and PCKh on MPII.

### 4.1. Ablation Study

We use our EEffPose-P0 for the ablation study, which is conducted on the COCO val set with an input size of 256 × 192. To study the effects of MEMe at different stages, we design eight ablation models that alternately use MEMe on the backbone, neck, and head. We report the metrics and their convergence curves for the analysis. All experiments are performed in the same configuration, except for the maximum learning epoch and the decay of learning rate due to the use of ReduceLROnPlateau, which reduces the learning rate when the validation AP stops improving. It is still a fair comparison and the learning rate decay curve can further reflect the convergence speed and difficulty of the model.

**Figure 8:**
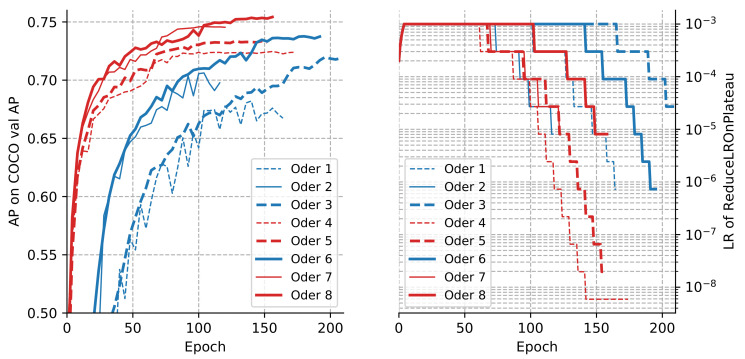
The convergence curves and LR self-adaption decay curves of ablation models.

MEMe-based Backbone (EEffNet). From Table 1, the following results can be seen: first, Order 2 gets a gain of 2.7 AP and 2.0 AR compared with Order 1, proving the effectiveness of using the EEffNet alone. Moreover, compared with Order 3, Order 6 increases the AP and AR scores by 2.1 and 1.8, showing that the MEMe backbone is still effective after using the TFNeck. Furthermore, Order 7 improves the AP and AR scores of Order 4 by 2.4 and 2.1, indicating that the MEMe backbone is still helpful on the basis of the FAHead. Finally, Order 8 shows 2.2 and 1.9 improvements on AP and AR scores than Order 5, demonstrating that the MEMe backbone can still make progress after using both the TFNeck and the FAHead. Meanwhile, a corroborating conclusion can be obtained from Figure 8 that Order 2, 6, 7 and 8 (solid lines) has higher AP scores during training than Order 1, 3, 4 and 5 (dotted lines). The above conclusions show that our enhanced backbone (EEffNet) can greatly increase the model capacity and improve performance.

MEMe-based Neck (TFNeck). From Table 1, the following results can be seen: first, Order 3 gets a gain of 3.8 AP and 1.9 AR compared with Order 1, proving the effectiveness of using the TFNeck alone. Moreover, compared with Order 2, Order 6 increases the AP and AR scores by 3.0 and 1.7, showing that the TFNeck is still effective after using the EEffNet. Furthermore, Order 5 improves the AP and AR scores of Order 4 by 0.9 and 0.8, indicating that the TFNeck is still helpful on the basis of the FAHead. Finally, Order 8 shows 0.7 and 0.6 improvements on AP and AR scores than Order 7, demonstrating that the TFNeck can still make progress after using both the EEffNet and the FAHead. Meanwhile, Figure 8 corroborates that Order 3, 6, 5 and 8 (thick lines) has steadier and more sustainable convergence than Order 1, 2, 4 and 7 (thin lines) instead of falling into local optima too soon. The above conclusions show that our enhanced neck (TFNeck) can maintain a stable and sustainable convergence and prevent falling into a local optimal situation.

MEMe-based Head (FAHead). From Table 1, the following results can be seen: First, Order 4 gets a gain of 4.5 AP and 2.7 AR compared with Order 1, proving the effectiveness of using the FAHead alone; Moreover, compared with Order 2, Order 7 increases the AP and AR scores by 4.2 and 2.8, showing that the FAHead is still effective after using the EEffNet. Furthermore, Order 5 improves the AP and AR scores of Order 3 by 1.6 and 1.6, indicating that the FAHead is still helpful on the basis of the TFNeck. Finally, Order 8 shows 1.9 and 1.7 improvements on AP and AR scores than Order 6, demonstrating that the FAHead can still make progress after using both the EEffNet and the TFNeck. Meanwhile, Figure 8 reveals that Order 4, 7, 5 and 8 (red lines) has faster and better convergence than Order 1, 2, 3 and 6 (blue lines). The above conclusions show that our enhanced head (EEffNet) can boost the model to get a fast and excellent convergence during training time.

**Figure 9:**
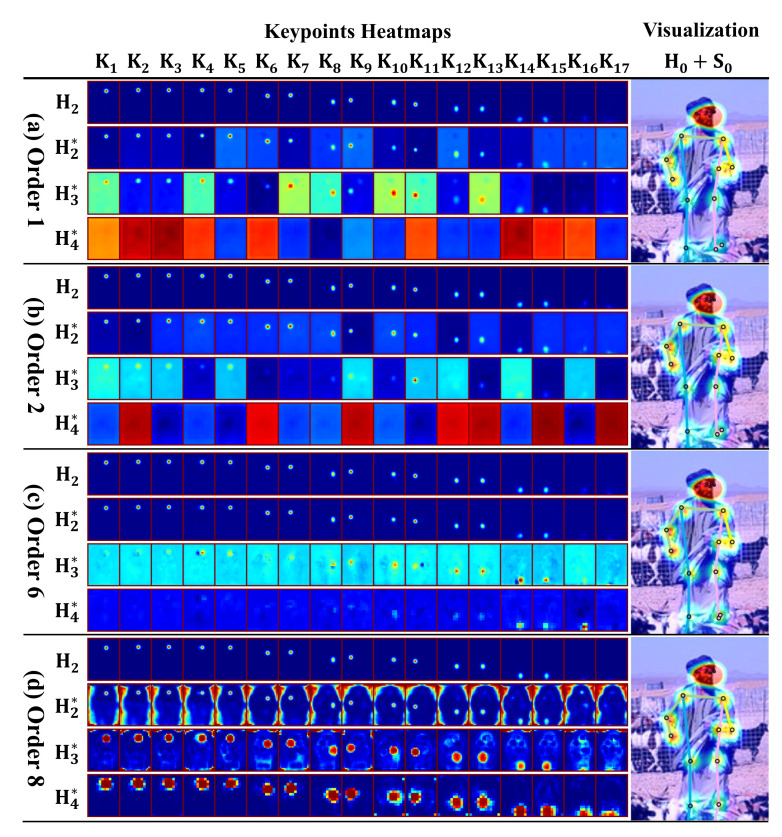
Visualization of the final outputs. (**a**) The outputs of Order 1, which means the baseline EffBase. (**b**) The outputs of Order 2 where the backbone is MEMe-based. (**c**) The outputs of Order 6 whose backbone and neck are both MEMe-based. (**d**) The outputs of Order 8, which is our final MEMe-based model EEffPose. K${}_{N}$ is the heatmap of keypoint while H${}_{J}^{*}$ and H${}_{2}$ are the multi-scale outputs, and the final one integrated as Equation (6) for (**a**–**c**) or Equation (7) for (**d**). For a better comparative observation, we upsampled H${}_{J}^{*}$ as the same scale as H${}_{2}$. The S${}_{0}$+H${}_{0}$ means S${}_{0}$ (the input image) overlaps with all keypoint heatmaps to demonstrate the detection results of the keypoints.

Visualization and Analysis. Figure 9 is used to clearly demonstrate the performance of our proposed three modules. Firstly, to show the effect of EEffNet, by the comparison of (a, b), the S${}_{2}$ and S${}_{3}$ of some keypoints, such as K${}_{14}$ and K${}_{15}$ obviously depict that our proposed EEffNet generates a more clear Gaussian Heatmap with a more powerful information extraction ability. Secondly, to show the performance of TFNeck, by the comparison of (a, b) and (c), (a, b)’s S${}_{4}$ of all keypoints shows the nearly blank heatmap (the bright color here is due to the pseudo-color conversion), which cannot extra a proper feature. While (c)’s K${}_{14}$ and K${}_{15}$ can get a better feature with our TFNeck, which proves the necessity of flexible information flow and weight-balanced feature fusion. Finally, to show the performance of FAHead, by comparison of (c, d), the phenomenon is that all scale outputs of (d) are the enhanced Gaussian heatmaps, just with different sizes, except K${}_{16}$ and K${}_{17}$. It proves that our FAHead, based on the cross attention mechanism, can generate better Gaussian heatmaps and reject the pseudo-shadow points, such as K${}_{16}$ and K${}_{17}$ in (a, c).

### 4.2. Comparisons to the State-of-the-Art

COCO val. In Table 2, for the EEffPose-P0 of 256 × 192, it achieves a score of 75.4 AP, which outperforms not only other small models, but also the big ones, such as HRNet-W48 with 256 × 192. For the EEffPose-P2 of 384 × 288, it achieves a 76.7 AP score and is higher than HRNet-W48, reaching the SOTA performance. Specifically, compared with the LiteHRNet-18/30, EEffPose-P0/P2 increases the AP by 10.6/6.3, with an acceptable increment in parameters. Similarly, compared with the DANet, EEffPose-P0/P2 increases the AP score by 4.4/3.3. Next, compared with other lightweight models, ShuffleNetV2 and MobileNetV2, both EEffPose-P0/P2 improve over 10 gains. Compared to large networks, such as HRNet, EEffPose-P0 can also achieve a higher AP score with a much lower model complexity. Especially, EEffPose-P0 with 256 × 192 has a higher AP score than that of HRNet-W48 with 384 × 288 using only 14% of its parameters. In Figure 1, it shows that our MEMe-based EEffPose reaches the optimal trade-off even with the best AP than others and enhances the lightweight model’s performance to cross the gap without too much pain.

COCO test-dev. Table 3 depicts the experimental results of our MEMe-based EEffPose and other methods. Compared with the small networks, such as LiteHRNet, DANet, MobileNet, our proposed EEffPose-P0/P2 reach the SOTA performance with an AP score of 74.2/75.9. Compared with the large networks in the same input resolution level, our proposed EEffPose has a higher AP score than Hourglass, CPN, HRNet, and SimpleBaseline, with a much lower computation cost succeeding in finding the best trade-off.

MPII val. Table 4 shows the results of our EEffPose and other models with the input of 256 × 256. EEffPose-P0/P2 get 90.38/90.84 PCKh@0.5, respectively, which outperform the large model, such as HRNet-W32/W48, and the small models, such as MobileNetV2, MobileNetV3, ShuffleNetV2 and LiteHRNet. Furthermore, our EEffPose-P2 can still make progress while HRNet-W48 cannot, due to the saturated dataset performance [9].

## 5. Conclusions and Discussions

In this paper, we proposed the mutually enhanced modeling method (MEMe) to enhance the lightweight model EffBase into a highly efficient EEffPose, which enhanced the original EffNet, BiPFN, FSHead into the mutually enhanced EEffNet, TFNeck, and FAHead, to fill the gap between the high accuracy of the large models and the low accuracy of the small models. Extensive experiments prove that our proposed EEffPose reaches the state-of-the-art, with low cost, and our MEMe plays a key role in this.

Moreover, our proposed MEMe is a general enhancing modeling method that can be applied to other lightweight models in human pose estimations, to improve their accuracy. Moreover, the MEMe can also be applied to many other vision tasks, such as segmentation, object detection, object tracking, etc., to improve accuracy with a low model complexity. The rise of HRNet brings the basic architecture of the multi-scale feature representation while our proposed MEMe not only fills the performance gap, but also leads to evolution among mutually enhancing multi-scale features.

## Figures and Tables

**Table 1:** The AP and AR of ablation models on the COCO val set with the input size of 256 × 192.

Order	MEMe-Based Backbone	MEMe-Based Neck	MEMe-Based Head	AP↑	AR↑
1	✗	✗	✗	67.8	75.7
2	✓	✗	✗	70.5	77.7
3	✗	✓	✗	71.6	77.6
4	✗	✗	✓	72.3	78.4
5	✗	✓	✓	73.2	79.2
6	✓	✓	✗	73.5	79.4
7	✓	✗	✓	74.7	80.5
8	✓	✓	✓	**75.4**	**81.1**

It is worth mentioning that Order 1 depicts our baseline EffBase and Order 8 represents our EEffPose. In MEMe-based Backbone: ✗ means original backbone (EffNet), while ✓ means our backbone (EEffNet). In MEMe-based Neck: ✗ means original neck (BiFPN), while ✓ means our neck (TFNeck). In MEMe-based Head: ✗ means original head (FSHead), while ✓ means our head (FAHead).

**Table 2:** Comparisons to the state-of-the-art on the COCO val set.

	Method	Backbone	Input Size	Params	GFLOPs	AP↑	AP .5	AP .75	AP (M)	AP (L)	AR↑
Small	LiteHRNet [16]	LiteHRNet-18	256 × 192	1.1 M	0.2	64.8	86.7	73.0	62.1	70.5	71.2
		LiteHRNet-30	384 × 288	1.8 M	0.7	70.4	88.7	77.7	67.5	76.3	76.2
	DANet [17]	DANet-72	256 × 192	3.4 M	1.0	71.0	89.6	78.1	67.6	76.2	76.6
		DANet-102	256 × 192	5.8 M	1.8	73.4	90.6	80.5	69.9	78.6	78.8
	LPN [40]	ResNet-152	256 × 192	7.4 M	1.8	71.0	89.2	78.6	67.8	77.7	76.8
	ShuffleNetV2 [14]	ShuffleNetV2	256 × 192	7.6 M	1.28	59.9	85.4	66.3	56.6	66.2	66.4
		ShuffleNetV2	384 × 288	7.6 M	2.87	63.6	86.5	70.5	59.5	70.7	69.7
	MobileNetV2 [11]	MobileNetV2	256 × 192	9.6 M	1.48	64.6	87.4	72.3	61.1	71.2	70.7
		MobileNetV2	384 × 288	9.6 M	3.33	67.3	87.9	74.3	62.8	74.7	72.9
	Ours	EEffPose-P0	256 × 192	8.98 M	4.94	75.4	90.5	82.6	71.7	82.2	81.1
		EEffPose-P2	384 × 288	16.7 M	23.0	**76.7**	90.5	**83.1**	**72.9**	**83.6**	**82.1**
Large	8-S Hourglass [6]	Hourglass	256 × 192	25.2 M	26.2	66.9	-	-	-	-	-
	CPN [7]	ResNet-50	256 × 192	27.0 M	6.20	68.6	-	-	-	-	-
	HRNet [9]	HRNet-W32	256 × 192	28.5 M	7.70	74.4	90.5	81.9	70.8	81.0	79.8
		HRNet-W32	384 × 288	28.5 M	17.3	75.8	90.6	82.7	71.9	82.8	81.0
		HRNet-W48	256 × 192	63.6 M	15.7	75.1	90.6	82.2	71.5	81.8	80.4
		HRNet-W48	384 × 288	63.6 M	35.4	76.3	**90.8**	82.9	72.3	83.4	81.2
	SimpleBaseline [8]	ResNet-50	256 × 192	34.0 M	8.90	70.4	88.6	78.3	67.1	77.2	76.3
		ResNet-152	384 × 288	68.6 M	35.6	74.3	89.6	81.1	70.5	79.7	79.7

**Table 3:** Comparisons to the state-of-the-art on the COCO test set.

	Method	Backbone	Input Size	Params	GFLOPs	AP↑	AP .5	AP .75	AP (M)	AP (L)	AR↑
Small	LiteHRNet [16]	LiteHRNet-30	384 × 288	1.8 M	0.70	69.7	90.7	77.5	66.9	75.0	75.4
	DANet [17]	DANet-72	256 × 192	3.4 M	1.0	70.5	91.8	78.2	67.5	75.0	76.7
		DANet-88	256 × 192	5.3 M	1.4	71.5	91.9	79.5	68.5	76.0	77.6
	LPN [40]	ResNet-152	256 × 192	7.4 M	1.8	71.0	89.2	78.6	67.8	77.7	76.8
	MobileNetV2 [11]	MobileNetV2	384 × 288	9.6 M	3.33	66.8	90.0	74.0	62.6	73.3	72.3
	Ours	EEffPose-P0	256 × 192	8.98 M	4.94	74.2	92.0	82.1	70.9	79.9	80.0
		EEffPose-P2	384 × 288	16.7 M	23.0	**75.9**	92.4	**83.4**	**72.4**	**81.9**	**81.3**
Large	8-S Hourglass [6]	Hourglass	256 × 192	25.2 M	26.2	66.9	-	-	-	-	-
	CPN [7]	Res-Inception	384 × 288	-	-	72.1	91.4	80.0	68.7	77.2	78.5
	HRNet [9]	HRNet-W32	256 × 192	28.5 M	7.70	73.5	92.2	81.9	70.2	79.2	79.0
		HRNet-W32	384 × 288	28.5 M	17.3	74.9	**92.5**	82.8	71.3	80.9	80.1
		HRNet-W48	256 × 192	63.6 M	15.7	74.2	92.4	82.4	70.9	79.7	79.5
		HRNet-W48	384 × 288	63.6 M	35.4	75.5	**92.5**	83.3	71.9	81.5	80.5
	SimpleBaseline [8]	ResNet-152	384 × 288	68.6 M	35.6	73.7	91.9	81.1	70.3	80.0	79.0

**Table 4:** Comparisons to the state-of-the-art on the MPII val set.

Model	Params	GFLOPs	PCKh@0.5↑
EfficientPose I [27]	0.7 M	1.67	85.2
HRNet-W16 [16]	1.3 M	0.72	80.2
LiteHRNet-18 [16]	1.1 M	0.27	86.1
LiteHRNet-30 [16]	1.8 M	0.42	87.0
EfficientPose II [27]	1.7 M	7.70	88.2
EfficientPose III [27]	3.2 M	23.4	89.5
EfficientPose IV [27]	6.6 M	72.9	89.8
ShuffleNetV2 [14]	7.6 M	1.70	82.8
MobileNetV2 [11]	9.6 M	1.97	85.4
HRNet-W32 [9]	28.5 M	10.25	90.3
HRNet-W48 [9]	63.6 M	47.23	90.28 ^*^
EEffPose-P0	8.98 M	6.59	90.4
EEffPose-P2	16.72 M	30.7	**90.9**

* is a re-implementing result for the unreleased result.

## Data Availability

The code of EEffPose will be released on GitHub before 1 July 2022: https://github.com/igodogi/EEffPose and can also be early available by contact if interested; for MSCOCO dataset: https://cocodataset.org; for MPII dataset: http://human-pose.mpi-inf.mpg.de/.
